# Infusion-related reactions to Rituximab: pathogenic mechanism and proposal of a new modified desensitization protocol

**DOI:** 10.1186/2045-7022-4-S3-P55

**Published:** 2014-07-18

**Authors:** Luis F Ramirez, Carlos Cañas, Gabriel Tobon, Fabio Bonilla, Manuela Olaya, Carlos Serrano

**Affiliations:** 1 Fundacion Valle del Lili, Colombia; 2 Fundacion Valle del Lili, Autoimmune Diseases Unit, Colombia; 3 Fundacion Valle del Lili, Allergy Unit, Colombia

## Background

Rituximab is a chimeric monoclonal antibody against the surface protein CD20 of B cells, used in the treatment of hematologic malignances and autoimmune diseases. Some patients develop hypersensitivity reactions after the infusion. It is unknown the percentage of these which are due to an IgE allergic mechanism. Also, there is not a standardized desensitization protocol that allows us to induce tolerance to this drug in patients.

## Methods

Patients with autoimmune diseases that have had a hypersensitivity reactions to rituximab and have needed a subsequent administration of the drug were included. Skin tests (prick and IDR) at a concentration of 1 mg/ml were performed on all patients and five controls. Afterwards, patients were hospitalized for 24 hours and a modified desensitization protocol with three different concentrations across a duration of nine hours was initiated. Variations in the infusion rate and/or skip steps were made in the following applications. Premedication with acetaminophen, hydrocortisone and loratadine was used in each desensitization.

## Results

16 patients were included between October 2012 and January 2014. 38 desensitization protocols were performed. Skin tests were positive in one patient only (IDR), and were negative in the rest of the patients and the healthy controls. None of the patients had a serious reaction during infusions and all of them tolerated the whole dose of the drug.

## Conclusions

Rituximab could be administered successfully and safely to all the patients. Most of the reactions were infusion-related (non allergic), probably explained by cytokine release. More studies are needed to validate our findings.

